# Opportunistic Gulls Infected by Antibiotic‐Resistant Bacteria Show Contrasting Movement Behaviour

**DOI:** 10.1002/ece3.71257

**Published:** 2025-04-15

**Authors:** Víctor Martín‐Vélez, Tomás Montalvo, Francisco Ramirez, Jordi Figuerola, Clara Morral‐Puigmal, Raquel Planell, Sara Sabate, Gerard Bota, Joan Navarro

**Affiliations:** ^1^ Institut de Ciències del Mar (ICM), CSIC, Passeig Marítim de la Barceloneta Barcelona Spain; ^2^ Agencia de Salud Pública de Barcelona Barcelona Spain; ^3^ Institut D'investigació Biomèdica Sant Pau (IIB SANT PAU) Barcelona Spain; ^4^ CIBER Epidemiología y Salud Pública (CIBERESP) Madrid Spain; ^5^ Estación Biológica de Doñana (EBD) CSIC Sevilla Spain; ^6^ Conservation Biology Group (GBiC) Landscape Dynamics and Biodiversity Program, Centre de Ciència i Tecnología Forestal de Catalunya (CTFC) Solsona Spain

**Keywords:** accelerometer, GPS, *Larus michahellis*, movement ecology, one health

## Abstract

The emergence, spread and potential zoonotic importance of pathogenic‐resistant bacteria (e.g., *Escherichia coli* ) has fuelled the research on epidemiology and vector movement dynamics. However, little is known about the effects that apparently asymptomatic carriage may have on host behaviour. Here, we analysed and compared movement patterns and habitat use (focused on the different risk of exposure to Antibiotic Resistance) of yellow‐legged gulls ( *Larus michahellis* ) carrying (*n* = 10) and not carrying (*n* = 29) Antibiotic‐resistant *Escherichia coli.* Using data from GPS devices coupled with accelerometers, we found evidence that individuals carrying resistant *E. coli* , although previously considered asymptomatic, had lower accumulated travelled distances and moved over smaller areas. Antibiotic resistance carriage may affect movement patterns to some extent, as in this case, potentially reducing pathogen dispersal over large areas.

## Graphical abstract

Our work addresses a major research gap in animal ecology and movement behaviour by investigating differences between infected and uninfected gull individuals with *Escherichia coli* . More specifically, we investigated differences in movement metrics by using high‐resolution GPS movement data.

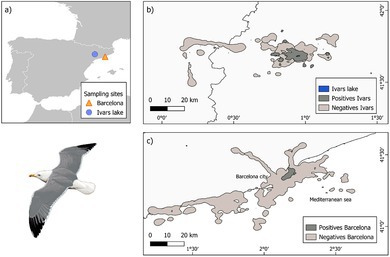

## 1 Introduction

Birds are considered important vectors for the dispersal of many zoonotic pathogens such as viruses (e.g., West Nile virus or Avian influenza; Koch et al. 2024; Yang et al. 2024) and bacteria (*Salmonella*, *Campylobacter* or *Escherichia coli* ; Lin et al. 2020). In many cases, birds are considered competent and asymptomatic hosts for these pathogens (Risely et al. 2018). However, little is known about the sub‐lethal effects that apparently asymptomatic infections may have on bird behaviour, ecology and life history (Benskin et al. 2009), which may ultimately influence the likelihood of pathogen dispersal itself (Risely et al. 2018).

The carriage of pathogens may impose physiological and behavioural constraints that may affect the host movements and the pathogen dispersal (Risely et al. 2018). Previous research on this topic has primarily examined the sub‐lethal effects of the influenza A virus on bird movement behaviour, yielding contrasting results. While some studies report no effects (Bengtsson et al. 2016), others have found altered local movements in mallards ( *Anas platyrhynchos* ) and reduced migration capacity in Bewick's swans ( *Cygnus columbianus bewickii* ; van Dijk et al. 2015; van Gils et al. 2007). However, the effects of zoonotic Antibiotic‐Resistant Bacteria (ARB) on bird movement behaviour remain largely unexplored and may be completely different from the effects of avian influenza (Benskin et al. 2009).

Antibiotic‐resistant bacteria are a global threat to human health, and their negative impact is expected to increase over the next years (Hernando‐Amado et al. 2019; Roca et al. 2015). Among ARB, *Escherichia coli* emerges as highly prevalent opportunistic enterobacteria in birds, mammals and humans, often considered commensal (Hubálek 2021). Many *E. coli* strains have zoonotic potential, and the emergence of antibiotic‐resistant clones in non‐pathogenic strains poses a risk due to the high genetic recombination rates within *E. coli* populations (Mukerji et al. 2020).

Different studies have determined a high incidence of antibiotic‐resistant *E. coli* (AR‐ *E. coli* ) in particular, in wildlife, including different scavenger species such as opportunistic gulls (Vergara et al. 2017; Martín‐Vélez, Navarro, et al. 2024), storks (Sacristán‐Soriano et al. 2024) or vultures (Blanco et al. 2020). Due to their high mobility and use of anthropic habitats, including dumps and other human infrastructures, gulls are hosts and potential vectors for the dispersal of ARB (Ahlstrom et al. 2021). However, our understanding of gulls as ARB vectors is limited, with little research on how ARB carriage affects their movement capacity. Sánchez‐Cano et al. (2024) previously reported an effect on travel distances in *E. coli* ‐carrying individuals in starlings. However, studies exploring the effect of *E. coli* carriage on other movement traits, as well as how these effects may be influenced by other stressors, remain lacking in birds. Indeed, the pathogenicity of most *E. coli* strains is often condition‐dependent (Collingwood et al. 2014), with sub‐lethal effects potentially emerging during high‐energy demanding periods such as reproduction.

Here, we investigate the potential impact of carriage of AR‐*E. coli* on the movement behaviour(distances, speeds, energy expenditure, areas) of breeding yellow‐legged gulls inhabiting urban and agricultural areas using GPS with accelerometer biologgers, a powerful approach to capture accurate behavioural information. In particular, we analyse and compare the movement patterns and habitat use between gulls carrying and not carrying AR‐*E. coli*, discussing the implications for pathogen dispersal.

## 2 Materials and Methods

### 2.1 Sampling Procedures

**FIGURE 1:**
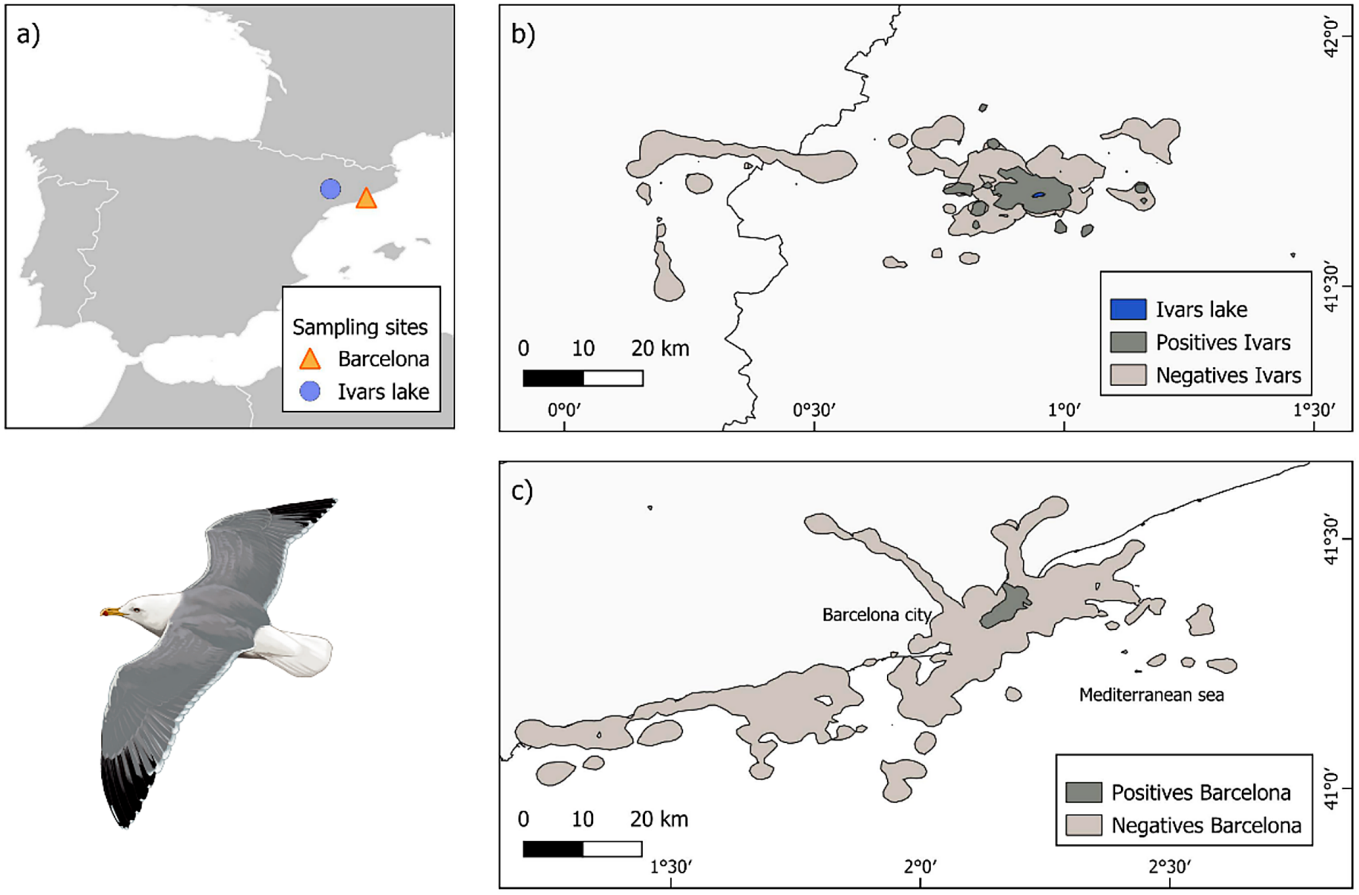
(a) Location of Barcelona and Ivars Lake sampling points. 95% utilisation distribution (UD) of infected (*n* = 10) and uninfected yellow‐legged gulls (*n* = 29) in (b) Barcelona and (c) Ivars shallow lake. Diagram of yellow‐legged gull made by Martí Franch.

This study was conducted during the yellow‐legged gull incubation period (April) of two consecutive breeding seasons (2022 and 2023) in the city of Barcelona and Ivars shallow lake (Catalonia, NE Spain, Figure 1A). The urban area of Barcelona hosts a population of yellow‐legged gulls of around 250–300 breeding pairs (Martín‐Vélez, Navarro, et al. 2024; Martín‐Vélez, Domingo, et al. 2024) that feed extensively on urban prey and human garbage (Martín‐Vélez et al. 2022; Vez‐Garzón et al. 2023). Ivars shallow lake hosts a population of 50–60 breeding pairs and is surrounded by agricultural habitats, including both irrigation and non‐irrigated crops, small towns and several irrigation ponds, livestock farms and small garbage dumps (Martín‐Vélez, Domingo, et al. 2024).

After a minimum of 10 days from the start of the incubation, 39 breeding adults (18 in Barcelona and 21 in Ivars) were caught at their nest (always three eggs) using a walk‐in wire mesh trap (Table S1). Immediately after the capture, each individual was kept in an individual clean cardboard box. After 10 min, we collected the faeces from the ground of the boxes with sterile swabs, stored in Cary‐Blair medium at 2°C–8°C. All yellow‐legged gulls were tagged with GPS‐accelerometer biologgers (OrniTtrack‐15 solar‐powered GPS‐GSM/GPRS tracker, Ornitela, Lithuania) recording the positions and accelerometer data (at 20 Hz) at 5‐min intervals. Biologgers were attached using a wing Teflon ribbon harness (see Thaxter et al. 2014). Biologgers and harness weighed less than 1.8% of the body mass of the birds (20 g for the devices versus 1062 ± 120 g [mean ± SD] for tracked gulls), less than the 3%–5% threshold suggested for seabirds (Passos et al. 2010). From three corporal feathers collected for each individual during the handling process, their sex was determined by molecular DNA determination (Dubiec and Zagalska‐Neubauer 2006).

### 2.2 ARB Determination

The presence of *E. coli* was analysed within 24 h at the ‘Agència de Salut Pública de Barcelona’ laboratory. Based on ARB assessments, we categorised individuals into two groups: positive (those carrying AR‐ *E. coli* ) and negative (those not carrying AR‐ *E. coli* ). Individuals were also tested for the detection of *Campylobacter* spp., *Salmonella* spp., *Y. enterocolitica* and *L. monocytogenes* following the ISO standard methods (see Martín‐Vélez, Navarro, et al. 2024). Faecal samples collected by sterile swabs from the 39 individuals captured were tested for the detection (only presence or absence) of extended spectrum beta lactamases (ESBL) and/or carbapenemase‐producing *E. coli* . The swab was directly plated in CHROMID ESBL agar (bioMérieux) and CHROMID CARBA SMART agar (bioMérieux) in order to isolate Enterobacteriaceae producing ESBL and/or carbapenemases, respectively. Burgundy red colonies were selected according to the manufacturer's instructions. Strains negative for the cytochrome oxidase enzyme were identified as *E. coli* . Some individuals were coinfected or tested positive to other pathogens (*Listeria monocytogenes, Campylobacter jejuni*, and *Salmonella* spp.), but due to the low prevalence (one infected per pathogen) were not considered for the study.

### 2.3 Movement Behaviour Analysis

GPS data accounted for 2022 and 2023 breeding periods, individuals were captured during late incubation and GPS data span for a 30‐day period, thus including also the chick‐rearing. Date, time, latitude, longitude, instantaneous speed and raw acceleration values (*A*_xe_, *A*_y_, *A*_z_) in g/1000 units were downloaded from each GPS device. Each gull location was assigned to a habitat by overlapping GPS data with land cover information. High‐resolution (1:25.000) information on land cover was obtained from the program SIOSE (Soil Information System of Spain, Instituto Geográfico Nacional, last update 2014). SIOSE categories were re‐classified into two overall categories associated to the potential risk of ARB exposure: ‘high‐risk habitat’ that considered agricultural/livestock habitats (Lopes et al. 2022; Martín‐Vélez, Navarro, et al. 2024) and dumps (Ahlstrom et al. 2021; Iwu et al. 2020), and ‘low‐risk habitat’ that included other habitats such as urban, industrial, beach, forest, sea harbour, waterbodies and sea. Although urban and industrial habitats are sometimes considered of ‘high risk’ for ARB, we considered them as ‘low’ as ARB exposures (mainly waste bins) are very discrete and would lead to an overestimation of ARB risks. We then estimated the total percentage of time spent in high‐ and low‐risk habitats by positive and negative to AR‐*E. coli*.

Each gull location was also assigned a behavioural state using the Expectation Maximisation binary Clustering (EMbC, Garriga et al. 2016) algorithm to classify the behaviours of gulls based on speed and turning angle. These variables are obtained from successive locations of the animal and are used to cluster each location into one of four behavioural categories: high velocity/low turn (HL), high velocity/high turn (HH), low velocity/low turn (LL), and low velocity/high turn (LH). HL is interpreted as ‘travelling/commuting’, HH as ‘extensive search’, LH as ‘intensive search’ and LL as ‘resting’ (Afán et al. 2019). The smoothing procedure (delta = 0.9) included in the package was applied to better account for the temporal associations among behaviours. We then calculated the time that every individual performs each behaviour and calculated the percentage of each individual based on the total spent time. We tested differences in percentage of time spent between behaviours, carriage and the interaction between both variables by carrying out a GLMM with a binomial error distribution (logit‐link) with individual as a random factor (Table S2).

We computed six movement metrics from GPS data (Table 1) to describe the daily movement of each GPS‐tagged gull. To avoid potential handling effects on movement due to capture, we excluded data from the first day after animal marking (Bengtsson et al. 2016). As we ignore the stage of the carriage process, we filtered the first 30 days (or for the time range available otherwise because of device failure; 29.4 and 29.5 days on average for carriers and non‐carriers respectively) which is the maximum reported shedding time that gulls still can be vectors for ARB (Franklin et al. 2020).

**TABLE 1:** Description and abbreviation of the six movement metrics analysed.

Movement metric	Explanation
Total cumulative distance travelled (*D*_tot_)	The sum of the lengths of the recorded trajectory measured in metres using great circle distances between all consecutive recorded locations
Maximum distance (*D*_max_)	The maximum distance between any two fixes of a bird during a certain sampling day
Mean speed (*V*_mean_)	Mean instantaneous speed in kilometres per hour per sampling day
Maximum speed (*V*_max_)	Maximum value of instantaneous speed in kilometres per hour on a certain sampling day
ODBA	Overall dynamic body acceleration (ODBA) summed and normalised per individual per sampling day
Area of Autocorrelated Kernel Density Estimation (AKDE)	The area that the animal used according to AKDE containing 95% all observed locations

To quantify the area used by gulls, we determined the utilisation distribution of 95% UD autocorrelated Kernel Density Estimation (AKDE) with package ctmm in R (Calabrese et al. 2016). To calculate AKDE area, we first used the functions ctmm.guess and ctmm.select based on the GPS database to fit the appropriate model to the data (Ornstein‐Uhlenbeck [OU] method was set by default) and generate the aKDE using a default setting, and determined the area for the resulting 95% UD. We also calculated additional variables from consecutive GPS positions (Haversine distance—spherical distance between geographic coordinates of GPS fixes and backwards time difference between GPS positions). To control for the potential effect of the reproductive duties (incubation or rearing the chicks), we calculated the percentage of time at the nest during the day (hereafter, nest attendance) based on the amount of accumulated time at the nest per sampling day in relation to the total of accumulated time. We determined nesting attendance by applying a buffer of 50 m around each nest and overlapping it with the GPS fixes database. We inspected each individual individually and removed from further analyses (e.g., linear mixed models) any individual that showed signs of breeding failure. This screening led to the exclusion of one positive individual and one negative individual (Figure S1).

To estimate energy expenditure, overall dynamic body acceleration (ODBA) was calculated from the raw accelerometer data recorded by the GPS devices (Wilson et al. 2006; Halsey et al. 2009). Each raw acceleration data (*A*_xe_, *A*_y_, *A*_z_) was smoothed through a high‐pass filter following Wilson et al. (2006) with the *tagtools* package (de Ruiter et al. 2023) to isolate a dynamic acceleration value from a static (*S*_x_, *S*_y_, *S*_z_) acceleration value (Bengtsson et al. 2016). The sampling rate was fixed at 20 Hz as it is the frequency that raw accelerometer values were recorded. The high‐pass filter cut‐off frequency was 3 s as it was reported as adequate for running means and smoothing data (Shepard et al. 2008). ODBA was calculated as the sum of all dynamic values of the three axes (*x*, *y*, *z*) in equation (1). The absolute ODBA values were then summed and normalised for all accelerometer values of all three dimensions within the segment to get the ODBA value associated with a discrete GPS fix (only GPS points with a segment with more than 20 raw acceleration values were used).

(1) $$\;\text{ODBA}=|A_{x}—S_{x}|+|A_{y}—S_{y}|+|A_{z}—S_{z}|$$

Parameters resulted in a single value describing the tri‐axial dynamic acceleration experienced, whereby larger values represent more movement of the body. All analyses were conducted in R 4.2.2 (R Development Core Team).

### 2.4 Statistical Analysis

We tested the association between carriage status (AR‐*E. coli* or not) and movement metrics (Table 1) using linear mixed models (LMM) and generalised linear models (GLMMs) with individuals as a random factor to account for repeated measures of the same individual and UTC date as a correlation factor nested with individuals to account for autoregressive correlation. To reduce the risk of collinearity between response variables, we used the *corrplot* R package (Wei et al. 2017) to calculate correlation (r) and excluded response variables (e.g., mean speed) with correlation higher than 0.7 (Figure S1). We considered the following predictive variables to include in the model: (1) sampling site, (2) infection status, (3) year, (4) sex, (5) body mass and (6) days since sampling as explanatory continuous covariates (Table S1). We also included (7) the number of GPS fixes per day and (8) nest attendance. The movement metrics were log‐transformed (when necessary) to satisfy normal residual distribution assumptions and standardised to facilitate comparison of estimates (Table S3). Quantification of movement metrics and all statistical analyses were performed with the package *nlme* (Pinheiro and Bates et al. 2024) in R 4.2.2. We evaluated the goodness of the fit of the model using pseudo‐*R* ^2^ as recommended for mixed models (Nakagawa and Schielzeth 2013) and followed a model selection procedure based on AIC using the function *dredge* from the package *Mumin* (Table S3; Barton and Barton 2015). To detect multicollinearity between covariables (Table S4) we used the Variance Inflation Factor (VIF) from the *car* package (Fox et al. 2012) and used a threshold of 5 to determine when multicollinearity was a concern (Thompson et al. 2017).

## 3 Results

Ten out of the 39 individuals sampled tested positive for AR‐*E. coli* (6 from Ivars and 4 from Barcelona). On average, positive individuals spent 55.5% of the time resting and 4.7% travelling, whereas negative individuals spent around 53% of the time resting and 6.6% of the time travelling. Positive individuals spent significantly more time resting and intensely searching and less time travelling than negative individuals (Table S2). There were differences between positives and negatives regarding the use of risky habitats (estimate = −0.13, *p* = 0. 02, Figure S3B), as positive gulls spent on average 10.4% and negative individuals 12.1% of the time in risky habitats.

**FIGURE 2:**
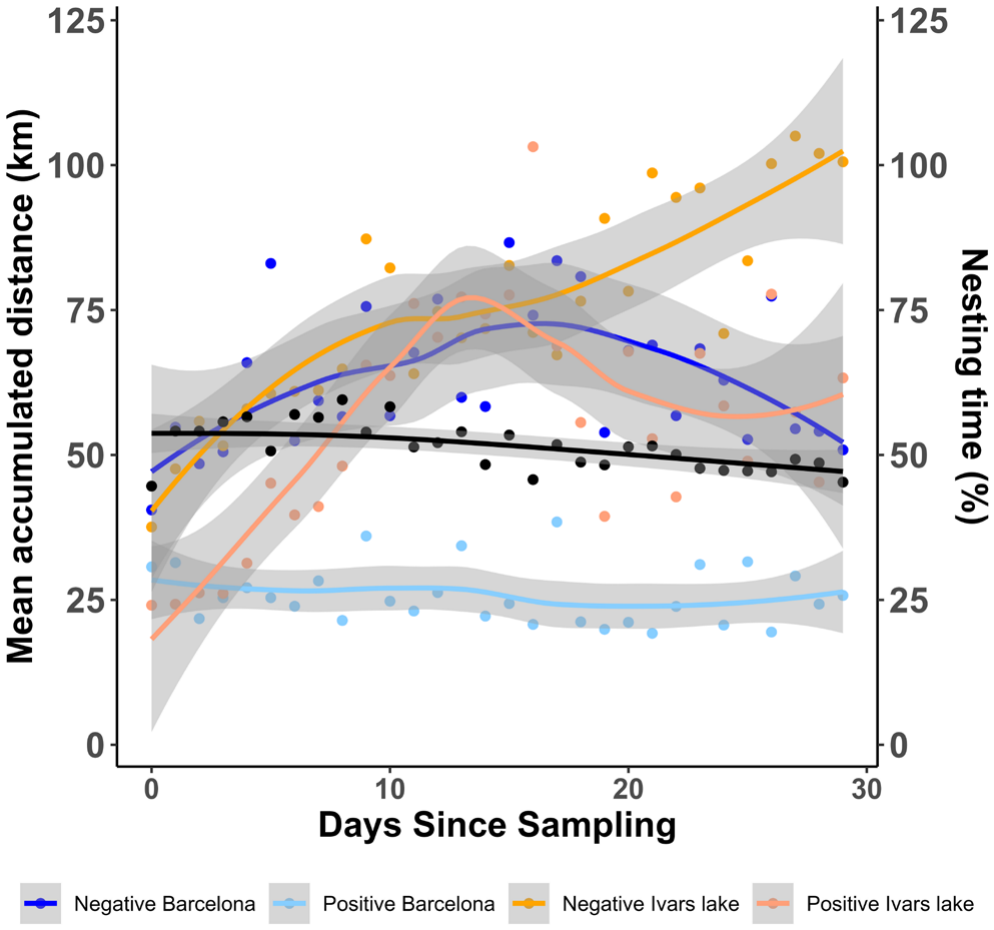
Change in accumulated distance (km) along days since sampling grouped by carriage status (negative to AR‐ *Escherichia coli* vs. positive to carry AR‐ *E. coli* ) and study site (Barcelona or Ivars Lake). The second axis (black line) shows the mean change in nest attendance along the sampling days. Coloured lines (blues for Barcelona and oranges for Ivars) show the LOESS adjustment (with span parameter by default and shading represents 95% CI).

Accumulated travelled distance (*D*_tot_: estimate = −1.77, *p* = 0.001; Tables 2 and 3, Figure 2), maximum daily travelled distance (*D*_max_: estimate = −0.94, *p* = 0.008) was lower in positive individuals (Tables 2 and 3). Positive individuals also attended the nest less (*D*_tot_: estimate = −0.57, *p* < 0.0001; *D*_max_: estimate = −0.19, *p* = 0.0001). Individuals from the Ivars Lake site travelled longer distances (*D*_tot_: estimate = 1.15, *p* < 0.0001; *D*_max_: estimate = −0.19, *p* = 0.0001) for accumulated distance (Figure 2) and maximum distance (Tables 2 and 3). When accounting for the interaction between the carriage and study site, positive individuals from Ivars showed higher travelled distances (*D*_tot_: estimate = 1.53, *p* < 0.0001; *D*_max_: estimate = 0.76, *p* = 0.007; Figure 2). Maximum speed (*V*_max_) was negatively related to nest attendance (estimate = −0.19, *p* = 0.0002) and significantly higher in positive individuals from Ivars Lake (estimate = 0.45, *p* = 0.009; Tables 2 and 3). Positive individuals covered smaller areas than the negatives (AKDE: estimate = −0.76, *p* < 0.001) (Table 2, Figure 1B,C).

**TABLE 2:** Mean and 95% CI (intervals between brackets) of the movement metrics used for comparison between positives (carriage of antibiotic‐resistant *E. coli* ) and negative yellow‐legged gulls.

Movement metric	Positives (carriage of AR‐*E. coli*)	Negatives (no carriage of AR‐*E. coli*)
Total cumulative distance travelled (*D*_tot_)	42.72 km (95% CI: 39.19, 46.34)	68.75 km (95% CI: 65.24–72.26)
Maximum distance (*D*_max_)	5.37 km (95% CI: 4.93–5.80)	6.22 km (95% CI: 5.90–6.54)
Mean speed (*V*_mean_)	3.35 km/h (95% CI: 3.05–3.66)	5.64 km/h (95% CI: 5.37–5.90)
Maximum speed (*V*_max_)	50 km/h (95% CI: 48.3–51.8)	56.03 km/h (95% CI: 55.3–57.3)
Mean normalised Overall Dynamic Body Acceleration (ODBA)	0.38 g (95% CI: 0.35–0.41)	0.33 g (95% CI: 0.31–0.34)
Area of Autocorrelated Kernel Density Estimation (AKDE)	37.5 km^2^ (95% CI: 30.5–44.6)	1435 km^2^ (95% CI: 339–2531)

**TABLE 3:** Summary results (estimate ± standard error) of the four linear mixed models (LMMs) analysing variation in (a) accumulated distance, (b) maximum distance, (c) mean speed, (d) maximum speed, (e) normalised ODBA (Overall Dynamic Body Acceleration) and (f) logarithmic area of AKDE (Autocorrelated Kernel Density Estimation). An individual was included as a random factor. Pseudo *R* ^2^ to test the goodness‐of‐fit is provided.

Metric	Pseudo‐*R* ^2^c	Intercept	Carriage	Site	N° fixes	Sampling day	Nest %	Year	Carriage*Site
Accumulated distance	0.42	−0.71 ± 0.13***	−1.77 ± 0.29 ***	0.80 ± 0.19 ***	—	—	−0.57 ± 0.05 ***	—	1.53 ± 0.38 ***
Maximum distance	0.39	0.37 ± 0.12**	−0.94 ± 0.21**	0.35 ± 0.13 *	−0.002 ± 0.0004 ***	—	−0.19 ± 0.04 ***	−0.29 ± 0.13 *	0.76 ± 0.26 **
Max speed	0.32	−0.59 ± 0.15***	−0.3 ± 0.19	0.64 ± 0.17*	—	—	−0.19 ± 0.04*	−0.44 ± 0.17*	0.91 ± 0.31 **
ODBA	0.44	−0.2 ± 0.14 ***	—	—	−0.002 ± 0.0001	—	—	—	—
AKDE	0.51	−0.21 ± 0.10	−0.76 ± 0.21 **	—	—	—	−0.40 ± 0.04 ***	—	—

* *p* ≤ 0.05.

** *p* ≤ 0.01.

*** *p* ≤ 0.001.

## 4 Discussion

Our results reveal that yellow‐legged gulls carrying AR‐ *E. coli* (i.e., individuals within the ‘positive’ group) moved over smaller areas and travelled less distances (individuals from Barcelona) than individuals not carrying *E. coli* (i.e., the ‘negative’ group). This has consequences for epidemiology of *E. coli* , as their potential for dispersal by birds may be limited by the smaller area used, especially in urban areas.

Carriage of AR‐ *E. coli* in gulls is typically asymptomatic, and minimal consequences would be expected by the sub‐lethal effects in terms of movement, except for some pathogenic strains that can cause enteritis and septicaemia, among other symptoms (Hubálek 2021). Despite showing no apparent symptoms, carriage of AR‐E. coli may shape the body condition of individuals, potentially limiting their daily movements, but not posing a risk for survival (Benskin et al. 2009). Although we did not assess physiological indicators or other resistant strains, all individuals showed no signs of illness and were breeding, indicating good body condition despite carrying AR‐ *E. coli* . Additional confounding factors that may influence our results include the age of the individuals (with immatures showing a higher prevalence of *E. coli* than adults; Martín‐Vélez, Navarro, et al. 2024) and the specific constraints associated with each breeding phase (e.g., incubation versus chick‐rearing). Here, we exclusively tagged breeding adults during the incubation period, thus minimising the potential role of these confounding factors. However, further studies should explore more in depth the sub‐lethal effects of pathogens in wildlife behaviour.

We found differences in the overall behavioural states (e.g., resting and travelling) between gulls carrying or not carrying AR‐E. coli. Previous studies found that adult yellow‐legged gulls in Barcelona spent around 20% of their time resting during the chick‐rearing period at sea (Gimeno et al. 2022). In contrast, this study observed a resting behaviour of 50%, likely due to the different demands of the incubation stage (Spelt et al. 2019). Regarding habitat use, we would expect individuals carrying AR‐ *E. coli* to frequent riskier habitats, such as dumps and agricultural–livestock areas, as these environments are known sources of ARB (Martín‐Vélez, Montalvo, et al. 2024). However, we found negative individuals even showing a higher use of risky habitats (Figure S2). Since we assessed habitat use after GPS deployment and testing for AR‐E. coli carriage status, we cannot rule out the possibility that the individuals may have used a different proportion of habitat or exhibited different behaviours before the capture and carriage. Alternatively, the carriage status may be more influenced by an individual's genetic/phenotypic condition or immune system status, rather than by using habitats with a higher risk of exposure to pathogens (Langager 2024). Furthermore, we used general habitats (such as urban and industrial) to explore habitat use, but AMR sources are normally located in more specific locations (e.g., rubbish bins, wastewater treatment plants, landfills), which may demand further detailed studies.

In our study, individuals carrying AR‐ *E. coli* present in Barcelona showed less travelled and maximum distances. We also found significantly lower values of maximum speed in individuals carrying AR‐ *E. coli* at Barcelona. Travelled distance also increased while the time spent at the nest decreased, likely due to the demands of chick‐rearing provisioning (Spelt et al. 2019). However, differences in age and breeding performance due to different moments of incubation may also determine travelling distances. Both carriage groups at both locations showed analogous shifts in maximum speed and travelled distances after day 10 (Figure 2), which may coincide with the end of incubation and the start of chick rearing (Spelt et al. 2019). However, considerable individual variation (especially in cities) in nesting stage timing and the associated requirements may have influenced these trends for positive individuals (Figure S1).

The smaller dispersal areas covered by ‘positive’ individuals during the shedding time may have potential implications for pathogen dynamics and could reduce the risk of transmission over larger areas (Duriez et al. 2023). Low movement patterns may, however, potentially increase the risk of transmission due to the accumulation and aggregation of individuals that carry AR‐ *E. coli* , especially in urban areas such as big cities like Barcelona (Martín‐Vélez, Navarro, et al. 2024). In fact, we cannot rule out the possibility that positive individuals more adapted to urban areas, which exhibit reduced movements, smaller travelled distances and areas, may have greater exposure to ARB sources, thereby increasing their carriage and potential vectoring of ARB (Martín‐Maldonado et al. 2020).

The use of new technologies such as GPS devices has advanced our understanding of bird movement and pathogen dynamics (van Dijk et al. 2015). This study is a first approach that combines the information provided by biologgers with the integration of epidemiology and movement ecology in pathogen dynamics. Acceleration and energy expenditure studies should be combined with more detailed technologies (e.g., video recording) for more detailed characterisation of animal behaviour (Sur et al. 2017). Future studies that determine the ARB loading in non‐breeding gulls with higher sample sizes combined with analysis of body condition and physiological status would help to further understand the temporal dynamics of infection in wildlife and their implications for potential pathogen transmission.

Although the *AR‐E. coli* prevalence observed in this study is notably higher than previously reported for the species in the same study area (i.e., Barcelona; Martín‐Vélez, Navarro, et al. 2024), we acknowledge that the small sample size in the ‘positive’ group limits the ability to draw strong conclusions about the sub‐lethal effects of *E. coli* infection. Understanding the effects of ARB in wildlife using GPS technology is challenging, particularly due to the difficulty in obtaining individuals carrying AR‐ *E. coli* at the time of sampling. Nevertheless, this study provides the first quantitative assessment of the sub‐lethal effects of AR‐ *E. coli* infection, though these results should be interpreted with caution.

## Author Contributions

**Víctor Martín‐Vélez:** conceptualization (equal), data curation (equal), formal analysis (equal), investigation (equal), methodology (equal), visualization (equal), writing – original draft (equal). **Tomás Montalvo:** methodology (equal), resources (equal), writing – review and editing (equal). **Francisco Ramirez:** methodology (equal), writing – review and editing (equal). **Jordi Figuerola:** conceptualization (equal), methodology (equal), writing – review and editing (equal). **Clara Morral‐Puigmal:** methodology (equal), resources (equal), writing – review and editing (equal). **Raquel Planell:** methodology (equal), resources (equal), writing – review and editing (equal). **Sara Sabate:** methodology (equal), resources (equal), writing – review and editing (equal). **Gerard Bota:** methodology (equal), resources (equal), writing – review and editing (equal). **Joan Navarro:** conceptualization (equal), funding acquisition (equal), project administration (equal), resources (equal), supervision (equal), writing – review and editing (equal).

## Ethics Statement

Fieldwork protocols were approved by the Ethics Committee of CSIC (REF: 28‐04‐15‐237) and the Catalunya Government (REF: AC/059‐23, SF/0068/23) in accordance with the Spanish and EU legislation on the protection of animals used for scientific purposes.

## Conflicts of Interest

The authors declare no conflicts of interest.

## Supporting information


Data S1.

Appendix S1.
**Figure S1.**Correlation plot between 6 response variables. Blue colours show positive correlation and red colours show negative correlation. Stronger the colour stronger the correlation.
**Figure S2.** Interindividual variation of nesting attendance between infected birds by antibiotic‐resistant *Escherichia coli* and non‐infected birds (blue and red colour respectively) along the 30 days after sampling that cover the shedding time for antibiotic‐resistant bacteria.
**Figure S3.** (a) Mean percentage (± S.E.) of time (%) that each yellow‐legged gull (grouped by its infection status by antibiotic‐resistant *Escherichia coli* ) spend on average performing each categorical behaviour: extensive search, intensive search, resting, travelling. (b) Percentage of time (%) that each individual (grouped by its carriage status by antibiotic‐resistant *Escherichia coli* ) spend on average in potential high risk habitat of ARB exposure high‐risk habitat or low‐risk habitat.
**Table S1.** Summary of individuals (ID) classified by sampling site (Barcelona, Ivars), infection status by antibiotic‐resistant *Escherichia coli* (if positive or not in bold), year (2022, 2023), sex (M = Male, F=Female), body mass (in grams) and sampling days that the GPS was transmitting until a maximum of 30 days.
**Table S2.** Model output (estimate, standard error, *Z*, *p* value) from beta regression (logit) model to test differences in time spent based on Carriage (positive or negative) and behaviour (extensive search, intensive search, resting, travel). Extensive search and negative carriage present the baseline of the intercept.
**Table S3.** Selected Linear Mixed Models (LMM) based on the AIC values (the AIC of the full model is also given) for the six metrics of movement: (a) logarithmic accumulated distance (*D*_tot_), (b) logarithmic maximum distance (*D*_max_), (c) maximum speed (*V*_max_), (d) mean normalised ODBA and (e) logarithmic area of Autocorrelated Kernel Density Estimation (AKDE). Gull identity (ID) was included as a random factor in all the models.
**Table S4.** Relation of Variance Inflation Factor (VIF) between the explanatory variables used in the models (Table S2) and the four metrics of movement (excluding ODBA and mean speed): logarithmic accumulated distance (*D*_tot_), logarithmic maximum distance (*D*_max_), maximum speed (*V*_max_), logarithmic area of Autocorrelated Kernel Density Estimation (AKDE).

## Data Availability

Data is uploaded as Supporting Information.
