# Supplementary material for: Opportunistic Gulls Infected by Antibiotic‐Resistant Bacteria Show Contrasting Movement Behaviour
Source: Ecol Evol. 2025 Apr 15;15(4):e71257. doi: 10.1002/ece3.71257 (PMC11997618; doi:10.1002/ece3.71257)
Supplement: Supplementary file 2 — Appendix S1. Figure S1.Correlation plot between 6 response variables. Blue colours show positive correlation and red colours show negative correlation. Stronger the colour stronger the correlation. Figure S2. Interindividual variation of nesting attendance between infected birds by antibiotic‐resistant Escherichia coli and non‐infected birds (blue and red colour respectively) along the 30 days after sampling that cover the shedding time for antibiotic‐resistant bacteria. Figure S3. (a) Mean percentage (± S.E.) of time (%) that each yellow‐legged gull (grouped by its infection status by antibiotic‐resistant Escherichia coli ) spend on average performing each categorical behaviour: extensive search, intensive search, resting, travelling. (b) Percentage of time (%) that each individual (grouped by its carriage status by antibiotic‐resistant Escherichia coli ) spend on average in potential high risk habitat of ARB exposure high‐risk habitat or low‐risk habitat. Table S1. Summary of individuals (ID) classified by sampling site (Barcelona, Ivars), infection status by antibiotic‐resistant Escherichia coli (if positive or not in bold), year (2022, 2023), sex (M = Male, F=Female), body mass (in grams) and sampling days that the GPS was transmitting until a maximum of 30 days. Table S2. Model output (estimate, standard error, Z, p value) from beta regression (logit) model to test differences in time spent based on Carriage (positive or negative) and behaviour (extensive search, intensive search, resting, travel). Extensive search and negative carriage present the baseline of the intercept. Table S3. Selected Linear Mixed Models (LMM) based on the AIC values (the AIC of the full model is also given) for the six metrics of movement: (a) logarithmic accumulated distance (D tot), (b) logarithmic maximum distance (D max), (c) maximum speed (V max), (d) mean normalised ODBA and (e) logarithmic area of Autocorrelated Kernel Density Estimation (AKDE). Gull identity (ID) was included [file ECE3-15-e71257-s002.docx]

**Supplementary**

**Table S1.** Summary of individuals (ID) classified by sampling site (Barcelona, Ivars), infection status by antibiotic resistant *Escherichia coli* (if positive or not in bold), year (2022, 2023), sex (M=Male, F=Female), body mass (in grams) and sampling days that the GPS was transmitting until a maximum of 30 days.

| ID | Site | Infection | Year | Sex | Body mass | Sampling days |
| --- | --- | --- | --- | --- | --- | --- |
| PBTP | Barcelona | Negative | 2022 | M | 1090 | 30 |
| PXJL | Barcelona | **Positive** | 2022 | F | 1150 | 30 |
| PXSF | Barcelona | Negative | 2022 | F | 850 | 30 |
| PXTC | Barcelona | **Positive** | 2022 | F | 920 | 30 |
| PXXY | Barcelona | **Positive** | 2022 | M | 1250 | 30 |
| PYDN | Barcelona | Negative | 2022 | M | 1125 | 30 |
| PYDX | Barcelona | Negative | 2022 | M | 860 | 29 |
| PYMB | Barcelona | Negative | 2022 | F | 900 | 30 |
| PZBA | Barcelona | Negative | 2022 | F | 980 | 28 |
| PZDB | Barcelona | **Positive** | 2022 | F | 930 | 30 |
| PYMA | Ivars | Negative | 2022 | M | 1000 | 30 |
| PYMF | Ivars | Negative | 2022 | M | 1200 | 30 |
| PYMH | Ivars | **Positive** | 2022 | M | 1260 | 30 |
| PYMK | Ivars | Negative | 2022 | M | 1200 | 30 |
| PYMT | Ivars | **Positive** | 2022 | F | 1040 | 30 |
| PYMU | Ivars | **Positive** | 2022 | F | 940 | 30 |
| PZBD | Barcelona | Negative | 2023 | M | 1075 | 30 |
| PZBF | Barcelona | Negative | 2023 | F | 860 | 30 |
| PZBS | Barcelona | Negative | 2023 | M | 1200 | 30 |
| PZBT | Barcelona | Negative | 2023 | F | 940 | 30 |
| PZCC | Barcelona | Negative | 2023 | M | 1140 | 30 |
| PZCT | Barcelona | Negative | 2023 | F | 950 | 30 |
| PZCW | Barcelona | Negative | 2023 | M | 1160 | 30 |
| PZCX | Barcelona | Negative | 2023 | F | 820 | 30 |
| PJWJ | Ivars | Negative | 2023 | F | 940 | 30 |
| PZCK | Ivars | Negative | 2023 | M | 1050 | 30 |
| PZCA | Ivars | **Positive** | 2023 | F | 920 | 27 |
| PZCB | Ivars | Negative | 2023 | F | 950 | 27 |
| PZCD | Ivars | Negative | 2023 | F | 990 | 30 |
| PZCF | Ivars | Negative | 2023 | M | 1100 | 24 |
| PZCJ | Ivars | **Positive** | 2023 | F | 830 | 30 |
| PZCL | Ivars | Negative | 2023 | F | 790 | 30 |
| PZCM | Ivars | **Positive** | 2023 | M | 1180 | 27 |
| PZCN | Ivars | Negative | 2023 | M | 1050 | 28 |
| PZCP | Ivars | Negative | 2023 | M | 1020 | 30 |
| PZCU | Ivars | Negative | 2023 | F | 960 | 30 |
| PZCV | Ivars | Negative | 2023 | F | 990 | 30 |
| PZCY | Ivars | Negative | 2023 | F | 950 | 30 |
| PZCZ | Ivars | Negative | 2023 | F | 980 | 30 |

**Table S2.** Model output (estimate, standard error, Z, p value) from beta regression (logit) model to test differences in time spent based on Carriage (positive or negative) and behaviour (extensive search, intensive search, resting, travel). Extensive search and negative carriage present the baseline of the intercept.

| Formula: percentage ~ carriage * behaviour + (1 \| ring_ID) | | | | |
| --- | --- | --- | --- | --- |
|  | **Estimate** | **S.E.** | **Z** | **p- value** |
| (Intercept) | -2.02292 | 0.01911 | -105.87 | < 0.0001 |
| Positive | -0.15829 | 0.0401 | -3.95 | < 0.0001 |
| Intense search | 1.04458 | 0.02372 | 44.03 | < 0.0001 |
| Resting | 2.18543 | 0.02301 | 94.98 | < 0.0001 |
| Travel | -0.65571 | 0.03016 | -21.74 | < 0.0001 |
| Positive*Intense | 0.27312 | 0.04915 | 5.56 | < 0.0001 |
| Positive*resting | 0.24869 | 0.04787 | 5.19 | < 0.0001 |
| Positive*travel | -0.16709 | 0.06521 | -2.56 | 0.0104 |

**Table S3.** Selected Linear Mixed Models (LMM) based on the AIC values (the AIC of the full model is also given) for the six metrics of movement: a) logarithmic accumulated distance (*D_tot_*), b) logarithmic maximum distance (*D_max_*), c) maximum speed (*V*max), d) mean normalized ODBA and e) logarithmic area of Autocorrelated Kernel Density Estimation (AKDE). Gull identity (ID) was included as a random factor in all the models.

| Most complete model: lmer (Metric~ year+ test + site + sampling day + sex + body mass + nest percentage +location*test+n fixes, random = ~ 1 \| ID, correlation = corAR1(form = ~ UTC_date \| ID)) |  |  |
| --- | --- | --- |
| Selected model: | **AIC_total model_** | **AIC** |
| a) lme (log *D_tot_* ~ test + site+ nest percentage+test*site, random = ~ 1 \| ID, correlation = corAR1(form = ~ UTC_date \| ring_ID)) | **2547.95** | **2508.2** |
| b) lme (log *D_max_* ~ test + site+ nest percentage + nº fixes +year, random = ~ 1 \| ID, correlation = corAR1(form = ~ UTC_date \| ID)) | **1899.26** | **1874.9** |
| C) lme (*V_max_* ~ test + site+ nest percentage+year, random = ~ 1 \| ID, correlation = corAR1(form = ~ UTC_date \| ID)) | **2556.27** | **2512.439** |
| D) glmmTMB (*ODBA* ~ test + site, random = ~ 1 \| ID, correlation = corAR1(form = ~ UTC_date \| ID)) | **1290.60** | **1275.798** |
| E) lme (log *AKDE* ~ test + nest percentage, random = ~ 1 \| ID, correlation = corAR1(form = ~ UTC_date \| ID)) | **2251.00** | **2227.278** |

**Table S4.** Relation of Variance Inflation Factor (VIF) between the explanatory variables used in the models (Table S2) and the four metrics of movement (excluding ODBA and mean speed): logarithmic accumulated distance (*D_tot_*), logarithmic maximum distance (*D_max_*), maximum speed (*V*max), logarithmic area of Autocorrelated Kernel Density Estimation (AKDE).

| **Model** | **Test** | **Sampling site** | **Year** | **Nº fixes** | **Nest percentage** | **Site*test** |
| --- | --- | --- | --- | --- | --- | --- |
| **Accumulated distance** | 2.442 | 1.437 | - | - | 1.187 | 2.906 |
| **Max. distance** | 2.791 | 1.489 | 1.372 | 1.145 | 1.25 | 2.99 |
| **Max. speed** | 2.81 | 1.516 | 1.27 | - | 1.239 | 3 |
| **AKDE** | 1.001 | - | - | - | 1.001 | - |


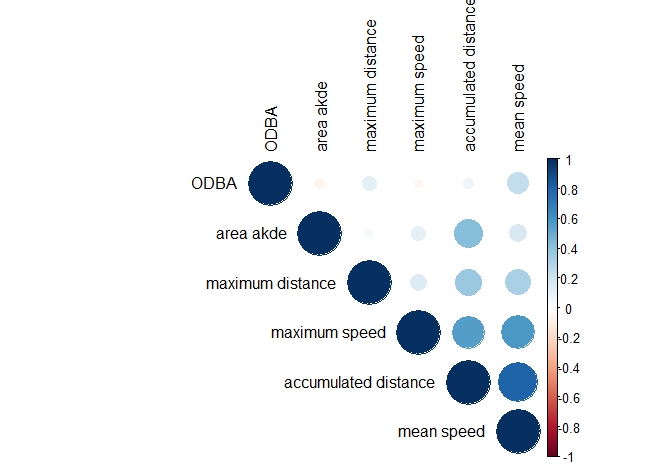


**Figure S1.** Correlation plot between 6 response variables. Blue colours show positive correlation and red colours show negative correlation. Stronger the colour stronger the correlation.


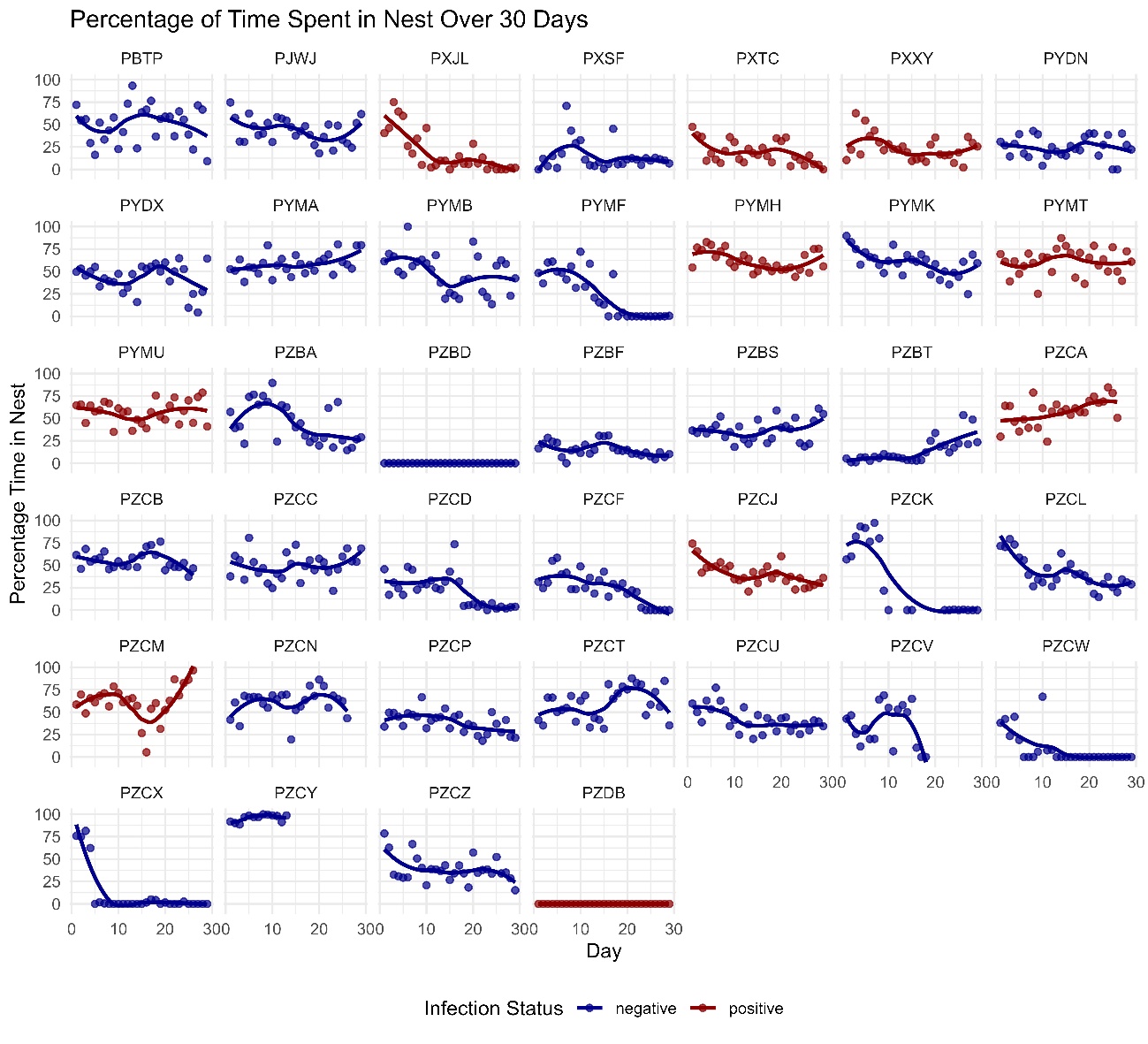


**Figure S2.** Inter individual variation of nesting attendance between infected birds by antibiotic resistant *Escherichia coli* and non-infected birds (blue and red colour respectively) along the 30 days after sampling that cover the shedding time for antibiotic resistant bacteria.

**Figure S3.** (a) Mean percentage (± S.E.) of time (%) that each yellow-legged gull (grouped by its infection status by antibiotic resistant *Escherichia coli*) spend on average performing each categorical behaviour: extensive search, intensive search, resting, travelling. (*b*) Percentage of time (%) that each individual (grouped by its carriage status by antibiotic resistant *Escherichia coli*) spend on average in potential high risk habitat of ARB exposure high risk habitat or low risk habitat.
